# EEG Error Prediction as a Solution for Combining the Advantages of Retrieval Practice and Errorless Learning

**DOI:** 10.3389/fnhum.2017.00140

**Published:** 2017-03-27

**Authors:** Ellyn A. Riley, Dennis J. McFarland

**Affiliations:** ^1^Aphasia Lab, Communication Sciences and Disorders, Syracuse UniversitySyracuse, NY, USA; ^2^National Center for Adaptive Neurotechnologies, Wadsworth Center, New York State Department of HealthAlbany, NY, USA

**Keywords:** aphasia, retrieval, errorless learning, EEG, predictive models

## Abstract

Given the frequency of naming errors in aphasia, a common aim of speech and language rehabilitation is the improvement of naming. Based on evidence of significant word recall improvements in patients with memory impairments, errorless learning methods have been successfully applied to naming therapy in aphasia; however, other evidence suggests that although errorless learning can lead to better performance during treatment sessions, retrieval practice may be the key to lasting improvements. Task performance may vary with brain state (e.g., level of arousal, degree of task focus), and changes in brain state can be detected using EEG. With the ultimate goal of designing a system that monitors patient brain state in real time during therapy, we sought to determine whether errors could be predicted using spectral features obtained from an analysis of EEG. Thus, this study aimed to investigate the use of individual EEG responses to predict error production in aphasia. Eight participants with aphasia each completed 900 object-naming trials across three sessions while EEG was recorded and response accuracy scored for each trial. Analysis of the EEG response for seven of the eight participants showed significant correlations between EEG features and response accuracy (correct vs. incorrect) and error correction (correct, self-corrected, incorrect). Furthermore, upon combining the training data for the first two sessions, the model generalized to predict accuracy for performance in the third session for seven participants when accuracy was used as a predictor, and for five participants when error correction category was used as a predictor. With such ability to predict errors during therapy, it may be possible to use this information to intervene with errorless learning strategies only when necessary, thereby allowing patients to benefit from both the high within-session success of errorless learning as well as the longer-term improvements associated with retrieval practice.

## Introduction

Individuals with aphasia commonly demonstrate deficits in lexical retrieval ability, which can manifest as difficulty naming objects and generating specific words in conversation. The severity of word retrieval impairment varies widely, and individuals present with unique error patterns; however, word retrieval impairment is typically observed across all aphasia subtypes. Given the pervasiveness of lexical retrieval impairments in persons with aphasia, improving object naming is a typical focus in speech and language therapy for this population. Many different treatment protocols for object naming have shown to effectively improve naming ability in persons with aphasia. Although the specifics of each treatment protocol differ, many fall under one of two general treatment philosophies – the retrieval approach or the errorless learning approach. Both approaches have evidence to support their efficacy, but results are mixed as to which works better. Recent work has suggested that naming improvement occurs with both approaches (reviewed in Fillingham et al., 2003), but that retrieval practice is needed for more lasting improvements (e.g., Middleton et al., 2015).

The errorless learning approach precludes the client from producing an error response; it provides as much support as necessary to ensure that the client does not produce an incorrect response. For example, when the client is presented with a picture, the clinician may provide the name of the item for the client to repeat. The errorless learning approach theorizes that by avoiding error production in the therapy task, only the correct neural connections will be formed and strengthened (Squires et al., 1997). Several studies have measured and compared outcomes from both errorless learning and retrieval approaches in the context of aphasia treatment. Findings indicate that, at best, errorless learning provides outcomes equivalent to those of errorful learning (Fillingham et al., 2003, 2005, 2006; McKissock and Ward, 2007), although clients tend to prefer the errorless learning approach (Fillingham et al., 2006).

Generally speaking, treatments based on the retrieval approach present some stimulus (e.g., a picture of an object), and the client is asked to provide the object’s name. When the client produces an error response, the clinician typically provides some specific feedback and the client attempts to name the object again. The retrieval approach operates on very different underlying principles – namely, that individuals recovering from aphasia will establish neural connections to the appropriate lexical item by forcing the brain to attempt retrieval from the lexicon, even if the response results in an incorrect production. Some have argued that although errorless learning and retrieval practice may appear to produce similar outcomes, the long-term benefits of retrieval practice outweigh those of errorless learning (reviewed in Middleton and Schwartz, 2012). Despite evidence that individuals with aphasia may inadvertently learn to produce incorrect responses during retrieval-based treatment (Middleton and Schwartz, 2013), retrieval practice may lead to more lasting treatment benefits (Middleton et al., 2015).

Rather than pitting these approaches against each other, though, it may be more beneficial to combine them and take advantage of both. One way of achieving this combined approach would be to develop a means of predicting the accuracy of the client’s response even before it is produced. Several studies have shown evidence of a relationship between specific EEG spectral features and behavioral response. Furthermore, evidence from unimpaired and epileptic patients suggests that pre-stimulus brain state can reliably predict performance on memory tasks; however, the relationship between EEG spectral features and behavioral performance on language tasks in persons with stroke and aphasia has not yet been established.

Many studies have provided evidence that brain state, identified physiologically, can be predictive of behavior. Evidence from electroencephalography (EEG), magnetoencephalography (MEG) and electrocorticography (ECoG) have shown significant correlations between changes in theta, alpha, and gamma oscillations as well as successful memory encoding and retrieval after stimulus presentation (Klimesch et al., 1997; Osipova et al., 2006; Sederberg et al., 2007). Electrical brain activity occurring during language tasks has also been reported, although relationships between brain activity and task performance are unclear and predictive models have not yet been investigated. Evidence from ECoG studies in patients with epilepsy have shown significant increases in gamma activity for picture naming, word reading (Wu et al., 2011), verb generation (Edwards et al., 2010), and lexical decision tasks (Tanji et al., 2005). These studies, however, looked solely for gamma activity and did not measure changes in other frequency ranges. Although these studies provide some evidence that electrical activity produced by the brain (in these cases, measured in the gamma frequency band) can provide some information for cortical mapping of language processing, they provide no evidence that gamma frequency changes are related to performance on any of these language tasks.

The wide range of tasks and results can make interpretation of these studies challenging, but some have used their findings to design experiments that more directly test such relationships between brain activity and performance. Drawing on prior evidence that changes in alpha and theta waves occurring *prior* to stimulus presentation can predict successful memory encoding and recognition (e.g., Fell et al., 2011; Merkow et al., 2014), Burke et al. (2015) designed an experiment in which the participant’s own EEG response was used to trigger stimulus presentation. The authors hypothesized that if words were only presented when the participant was in an optimal brain state for learning, memory encoding and recall would improve. Results were somewhat mixed, showing better memory performance in this condition for some participants but not others. Although the response was not reliable across all participants, results showed that the number of sessions in which memory performance was enhanced by this method was greater than chance. In a similar study, Salari and Rose (2016) found more reliable memory improvements using the same approach, although these variations in memory performance were shown to be related to the amount of pre-stimulus beta activity.

Currently, there is not enough evidence in the literature to extract a single overarching pattern regarding the relationship between the brain’s electrical activity and cognitive behavioral performance or ability; however, from these studies, some observations can be made that may help to shape future research. Many of these tasks are lumped together into categories of “memory” or “language” tasks, but the fact that two different memory tasks elicit very different brain responses suggests that EEG response may be specific to the task itself. Furthermore, these responses may be specific not only to the task but also to the individual. Results from Burke et al. (2015) and Salari and Rose (2016) suggest that electrical activity can and does vary across individuals but can still result in similar behavioral outcomes. Research thus far has investigated brain/behavior relationships in unimpaired individuals and patients with epilepsy, but the relationship between EEG spectral features and behavioral performance on language tasks in persons with stroke and aphasia has not yet been established. If we can identify a significant relationship between EEG features and behavioral performance within an individual with aphasia, we can then use this information to create a tool that would provide client-specific predictions about task performance during speech and language therapy.

The aim of this study was to determine if we could generate a statistical model that would reliably predict participant response accuracy using the spectral features from EEG. More specifically, we aimed to determine whether EEG spectral features could be used to predict (1) accuracy and (2) error correction in persons with aphasia during a picture naming task. Regarding accuracy, we hypothesized that (1) EEG spectral power would significantly correlate with accuracy and (2) scores generated from the statistical model for each participant would significantly correlate with observed scores. Regarding error correction, we hypothesized that (1) EEG spectral power would significantly correlate with error type and (2) scores generated from the statistical model for each participant would significantly correlate with observed scores.

## Materials and Methods

### Participants

This study was approved by the Syracuse University Institutional Review Board and all participants gave written informed consent in accordance with the Declaration of Helsinki. For this study, participants were recruited from the aphasia therapy group at the Gebbie Speech and Hearing Clinic, the in-house speech and language clinic at the first author’s academic institution. Individuals were initially selected for participation based on their interest in participating and self-reported difficulty with naming or word-finding following stroke. As this study was intended to investigate the feasibility of using EEG to predict errors, we did not administer language testing prior to collecting EEG data but relied on clinician and client report of naming difficulty to identify eligible participants. In order to confirm reported naming difficulties, baseline picture naming accuracy was obtained for 300 items during the first test session (see **Table 1**). Error rates for participants ranged between 5 and 74% and seven of eight participants demonstrated naming errors for more than 25% of their responses.

**Table 1:** Participant demographic and language testing information.

Participant	Age	Years education	Sex	Handedness	Time post-stroke	WAB-R AQ	WAB-R Classification	Baseline picture naming accuracy
1501	70	17	Male	Right	2 years, 6 mo.	90.6	Anomic	82%


1503	46	15	Male	Right	3 years, 1 mo.	65.5	Broca’s	26%


1601	56	17	Male	Right	4 years, 3 mo.	78.5	Conduction	27%


1602	64	13	Male	Right	9 years, 1 mo.	73.9	Transcortical Motor	72%


1603	54	17	Male	Left	2 years	46.6	Broca’s	32%


1605	59	15	Male	Right	3 years, 8 mo.	94.2	Anomic	78%


1606	33	19	Female	Right	8 mo.	95.6	Anomic	95%


1607	79	13	Female	Right	1 year, 4 mo.	95.4	Anomic	72%




*WAB-R, Western Aphasia Battery-Revised; AQ, Aphasia Quotient.*

Six males and two females with aphasia completed this study (see **Table 1** for all demographic and language testing information). One participant (1604) dropped out during the first EEG session due to extreme frustration with the task. Participants ranged in age from 33 to 79 years (*M* = 57.6 years), were all pre-morbidly right-handed (except for participant 1603), and minimally completed a high school education (*M* = 15.8 years, range 13–19 years). All participants reported having a single, left-hemisphere stroke (except participant 1603, who had a right-hemisphere stroke) and time post-onset ranged from 8 months to 9 years, 1 month (*M* = 3 years, 3 months). The *Western Aphasia Battery-Revised* (WAB-R; Kertesz, 2007) was administered to all participants (except participant 1604) in a single session either prior to the first EEG session (participants 1501, 1503) or following the last EEG session (participants 1601, 1602, 1603, 1605, 1606, and 1607). Participants represented a range of aphasia severities, with a mean Aphasia Quotient of 80 (range 65.5–95.6). Using the WAB-R aphasia classifications, four participants (1501, 1605, 1606, and 1607) were classified as anomic, two (1503 and 1603) as Broca’s, one (1601) as conduction, and one (1602) as transcortical motor.

### EEG Equipment and Software

EEG was recorded with 9-mm tin electrodes embedded in a cap (ElectroCap, Inc.) at 16 scalp locations according to the 10–20 system of Jasper (1958). The electrodes were referenced to the right ear, and their signals were amplified and digitized at 256 Hz by g.USB amplifiers. BCI operation and data collection were supported by the BCI2000 platform (Schalk et al., 2004). BCI2000 is an open-source framework (Schalk et al., 2004) designed for real-time signal processing.

### Picture Stimuli Selection for Naming Task

Picture stimuli for this study were obtained from the Bank of Standardized Stimuli (BOSS; Brodeur et al., 2010, 2014). BOSS is a database of approximately 1,400 photographs of living and non-living objects, many of which have been normed on control participants for object name, category, familiarity, visual complexity, object agreement, viewpoint agreement, and manipulability. Photograph stimuli in this database are available in color, grayscale, blurred, or scrambled forms.

From the total of 1,469 items in the database, we excluded any photographs that received less than 20% naming agreement or had a mean familiarity rating of less than 75% (3.75 out of 5). From the remaining 1,064 items, 900 were then selected by the first author and two research assistants, who examined and rated them, eliminating any that were determined to be unclear (e.g., extreme close-ups) or too similar to another in the set (e.g., two photos of similar-looking jewelry). Each color photograph in the final set was unique, but some item names were repeated. For example, an alarm clock and a grandfather clock were included in the picture set, both of which could be correctly named as “clock,” but we were careful not to repeat item names within each session. Each item in the final set of 900 pictures was assigned a value by a random number generator, and items were sorted by these values and split into nine sets of 100 photographs. These sets were then checked for repeat names, and if a repeat name was discovered within one of the lists, it was traded with an item from another list.

### Experimental Testing Procedure

Once an individual consented to participate, the participant was scheduled to come into the Syracuse University Aphasia Lab for three EEG data collection sessions at his/her convenience. The amount of time between sessions varied across participants, with a mean of 11.75 days between sessions 1 and 2 (*SD* = 12.89) and a mean of 6.63 days between sessions 2 and 3 (*SD* = 3.74). During each EEG data collection session, participants were administered three of the photo stimuli sets: sets 1, 2, and 3 were administered in session 1; sets 4, 5, and 6 in session 2; and sets 7, 8, and 9 in session 3. Participants were given opportunities to take two breaks within each session; the first opportunity was offered after 1/3 of the items had been presented, and the second after 2/3 had been presented.

At the start of each session, the participant was seated in a stable chair in front of a computer monitor. In the first session, a trained research assistant measured the distance from the nasion to the inion to determine correct placement for the Fz electrode (i.e., 30% of this measurement, as standard in the 10/20 system for EEG setup). Once the electrical signal was stable, the research assistant started stimulus presentation using BCI2000 software. Participants were instructed to name the object displayed on the computer monitor as best they could, even if they were unsure. Each photograph was displayed for 10 s, followed by a maximum of 5 s between trials. The participant’s response was immediately scored for accuracy and error type by the research assistant administering the task. Once the research assistant assigned a score via buttons pressed on a numeric keypad, the experiment advanced to the next item. Video recordings of these sessions were obtained to check scoring reliability. The first author reviewed and scored one random session for each participant. Scoring reliability was 98% for accuracy and 89% for error type.

### Scoring System

The scoring system we used for this study was loosely based on the criteria used by Schwartz et al. (2016) for classifying errors. Our system consisted of 10 categories, each of which was assigned a numeric value on the response keypad (see **Table 2** for scoring categories and operational definitions).

**Table 2:** Response classifications and operational definitions.

Classification	Operational definition
No response	Participant does not respond within the 10 s when the picture is displayed
Correct	Noun matches target (allow for incorrect number marking, e.g., mouse/mice, cats/cat)
Self-corrected fragment	Self-interrupted response consisting minimally of CV or VC sequence, NOT repeated first sound of target, results in correct response
Fragment, Incorrect Response	Self-interrupted response consisting minimally of CV or VC sequence, NOT repeated first sound of target, results in INCORRECT response of any type
Semantic Error	Noun that conveys conceptual mismatch in form of: Category coordinate (trumpet/tuba), Thematic associate (pirate/treasure), Incorrect but related superordinate or subordinate (apple/vegetable; shoe/slipper)
Self-corrected semantic error	Initial production of semantic error, but correct response produced before 10 s time window elapses
Circumlocution	Participant describes the target but does not correctly name it
Phonological/Neologistic Error	Error that does not meet criteria for fragment or semantic error, includes non-words and real words of any category (nouns, verbs, adjectives, etc.)
Self-corrected phonological/Neologistic error	Initial production of phonological/neologistic error, but correct response produced before 10 s time window elapses
Perseveration	Participant repeats previous words or sound from previous target, results in an incorrect response


### Data Analysis

Looking only at the behavioral data, the number and percentage of correct and incorrect responses were calculated for each participant and session. For these, the initial accuracy calculations, no-response and self-corrected responses were counted as incorrect. Response percentages were then further broken down into specific error types for each participant and session.

EEG signals from the 16 scalp electrodes were subjected to a 40th order autoregressive spectral analysis (McFarland and Wolpaw, 2008). Amplitudes for 4-Hz-wide spectral bands from 6 to 29 Hz (i.e., 6–9, 10–13, 14–17, 18–21, 22–25, and 26–29 Hz) were computed for 1000 ms sliding windows that were updated every 62.5 ms. Next, the trial average of each potential feature (i.e., amplitudes for 4-Hz bands for electrodes Fz, Cz and Pz; F3, Fz, F4, C3, Cz, C4, P3, Pz, and P4 for 9-channel analysis) for the period between presentation of the stimulus and immediately prior to the subject’s response served as the dependent variable in regularized multiple regression models that predicted naming performance. For the regression analysis, we used the glmnet package from R (Friedman et al., 2010) with regularization (elastic net). The elastic net solves for the vector of regression weights using the formula:

$$\beta =\text{argmin}\left({\left\|y-X\beta \right\|}^{2}+\lambda_{2}{\left\|\beta \right\|}^{2}+\lambda_{1}{\left\|\beta \right\|}^{1}\right)$$

where *β* is the vector of regression weights, *y* is the vector of values of the dependent variable, *X* is the matrix of predictors and λ_2_ and λ_1_ are penalties on the regression weights. The λ_2_ penalty minimizes the sum of the squared regression coefficients and serves to smooth these values as in ridge regression. The λ_1_ penalty minimizes the sum of the absolute values of the regression coefficients and tends to force many of the coefficient values to 0 (i.e., a sparse solution), resulting in single step feature selection. The elastic net algorithm simultaneously optimizes the value of both penalty terms using embedded cross-validation of the training set data (Friedman et al., 2010). The elastic net is preferable to ordinary least-squares regression since it tends to better generalize to novel data and produces simpler models (Zou and Hastie, 2005).

The data recorded during sessions 1 and 2 served as the training set that was used to compute regression weights. The data from session 3 served as the test set that was used to evaluate the generalization of the models based on new data. This generalization was evaluated by the Pearson’s *r*-value between predicted and observed values.

## Results

### Accuracy and Error Types

Accuracy on the task ranged from 26 to 94% with a mean of 61.25% across all participants (see **Table 3** for individual participant data). Error patterns differed for individual participants, although some general error patterns can be noted. Two of the participants (1501, 1503) produced relatively equivalent proportions of semantic and phonological/neologistic errors, whereas the remaining six (1601, 1602, 1603, 1605, 1606, 1607) produced a higher proportion of semantic errors as compared to phonological/neologistic errors. Successful self-corrections were rare for all participants (≤7% of all trials), with the exception of participant 1503, who was able to self-correct errors approximately 11% of the time.

**Table 3:** Frequency of response type for each participant.

Response Type	Participant #							
	
	1501	1503	1601	1602	1603	1605	1606	1607
Correct	84%	26%	26%	75%	34%	78%	94%	74%
No response	3%	14%	26%	2%	24%	3%	0%	7%
Self-corrected fragment	1%	1%	0%	0%	0%	0%	0%	0%
Fragment, incorrect	0%	6%	1%	0%	1%	0%	0%	0%
Semantic error	5%	20%	19%	15%	26%	8%	3%	11%
Self-corrected semantic error	0%	0%	1%	5%	0%	2%	2%	3%
Circumlocution	0%	2%	23%	1%	1%	0%	1%	2%
Phonological/Neologistic error	6%	20%	2%	0%	11%	3%	0%	1%
Self-corrected phonological/Neologistic Error	1%	11%	1%	1%	1%	5%	0%	2%
Perseveration	0%	0%	0%	0%	1%	0%	0%	0%


Although error patterns differed widely across participants, within-participant accuracy remained consistent across experimental sessions. A repeated measures ANOVA test was used to compare the number of pictures named correctly across the three sessions. Mauchly’s test indicated that the assumption of sphericity was met, *X*^2^(2) = 4.95, *p* = 0.084, and results of the ANOVA indicated that accuracy (i.e., total number correct) did not significantly differ across sessions, *F*(2,14) = 0.435, *p* = 0.656. Repeated measures ANOVA tests were also conducted for the three most frequent error types: no-response, semantic errors, and phonological/neologistic errors. For no-response errors, Mauchly’s test indicated that the assumption of sphericity was met, *X*^2^(2) = 4.09, *p* = 0.129, and results of the ANOVA indicated that the number of no-response trials did not significantly differ across sessions, *F*(2,14) = 1.85, *p* = 0.193. For semantic errors, Mauchly’s test indicated that the assumption of sphericity was met, *X*^2^(2) = 1.35, *p* = 0.509, and results of the ANOVA indicated that the number of semantic error trials did not significantly differ across sessions, *F*(2,14) = 1.60, *p* = 0.237. For phonological/neologistic errors, Mauchly’s test indicated that the assumption of sphericity was violated, *X*^2^(2) = 8.04, *p* = 0.018; therefore, Greenhouse–Geisser corrected tests are reported (ε = 0.575). Results of the phonological/neologistic error ANOVA indicated that the number of phonological/neologistic trials did not significantly differ across sessions, *F*(1.15,8.05) = 0.241, *p* = 0.670.

### EEG Results

Regression coefficients for training and test data for the three-channel analysis are reported in **Table 4** and correlations are reported in **Table 5**. As hypothesized, during the naming task, EEG spectral power correlated significantly with accuracy in most participants with aphasia. For this first model, we attempted to predict the classification of responses into two categories: correct and incorrect. Only responses matching the target on the first production attempt were scored as correct. For this model, “incorrect” included all other scoring categories (e.g., no-response, semantic errors, self-corrected semantic errors). When using the three-channel model with penalties, there were significant correlations between spectral power and accuracy in the training sessions for all participants except 1602. Furthermore, the scores generated from the model for all but one participant (1602) significantly correlated with the observed scores in the naming task. A subsequent analysis including nine channels did not significantly improve accuracy prediction during the test phase.

**Table 4:** Regression coefficients and degrees of freedom for training and test data.

Feature	1501	1503	1601	1602	1603	1605	1606	1607
Intercept	0.5437	-0.0742	-0.8258	0.7295	0.3214	0.6840	0.7284	0.8471


8 Hz Fz			0.0347	0.0001		-0.0092	0.0050	-0.0002


8 Hz Cz		-0.0125						
8 Hz Pz								
12 Hz Fz		-0.0284				0.0168	0.0029	-0.0029
12 Hz Cz	0.0101			0.0003		-0.0190	0.0086	-0.0041
12 Hz Pz		0.0962	0.0124			0.0237	0.0003	
16 Hz Fz		-0.0107	0.0240					
16 Hz Cz						-0.0029		
16 Hz Pz						-0.0378		
20 Hz Fz		-0.0064				-0.0105	-0.0014	
20 Hz Cz						0.0649		
20 Hz Pz								
24 Hz Fz								
24 Hz Cz	0.0278	0.0159			0.0352	-0.0058		0.0513
24 Hz Pz								-0.0147
28 Hz Fz	0.0060	0.0062				0.0524		-0.0053
28 Hz Cz		0.0428	0.0475			-0.0563		
28 Hz Pz		-0.1091			-0.0381			-0.0560
df train	3/596	8/590	3/585	1/597	1/596	10/588	4/593	6/592
df test	3/296	8/290	3/295	1/297	1/297	10/288	4/295	6/292


*Features with 0 weights are left blank.*

**Table 5:** Correlations for model predicting accuracy and error correction.

Participant	Accuracy			Error Correction	
		
	Train, 3-channel	Test, 3-channel	Test, 9-channel	Train, 3-channel	Test, 3-channel
1501	0.2148***	0.2456****	0.2535****	0.1392*	0.1950**
1503	0.4673****	0.2589****	0.3098****	0.4176****	0.1607**
1601	0.4710****	0.4570****	0.5085****	0.2296***	0.1234*
1602	0.0409	-0.0516	NA	NA	NA
1603	0.2442***	0.1244*	0.2037***	0.1531*	0.0627
1605	0.3081****	0.2588****	0.2781****	0.2550****	0.2113***
1606	0.1989**	0.2044***	NA	0.1701**	0.2142***
1607	0.3921****	0.2980****	0.3433****	0.2538****	0.1332*


*^∗^*p* < 0.05; ^∗∗^*p* < 0.01; ^∗∗∗^*p* < 0.001; ^∗∗∗∗^*p* < 0.0001.*

Similar results were generated when error correction was added as a predictor, although the correlations were not as strong as for the accuracy-only model. For this second model, we attempted to predict the classification of responses into three categories: correct, incorrect, and self-corrected. Only responses matching the target on the first production attempt were scored as correct. For this model, “incorrect” included only scoring categories that started out as errors and were not corrected, including fragment errors, semantic errors, and phonological/neologistic errors. The classification “self-corrected” included scoring categories which started out as errors but were independently corrected by the participant before the end of the trial. These included self-corrected fragments, self-corrected semantic errors, and self-corrected phonological/neologistic errors. Trials with no response, circumlocutions, and perseverations were not included in this second model. When using the three- channel model with penalties, EEG spectral power significantly correlated with error category in all participants except 1602. Furthermore, the model’s predicted error classifications for six of the participants significantly correlated with observed error classifications in the naming task.

**FIGURE 1:**
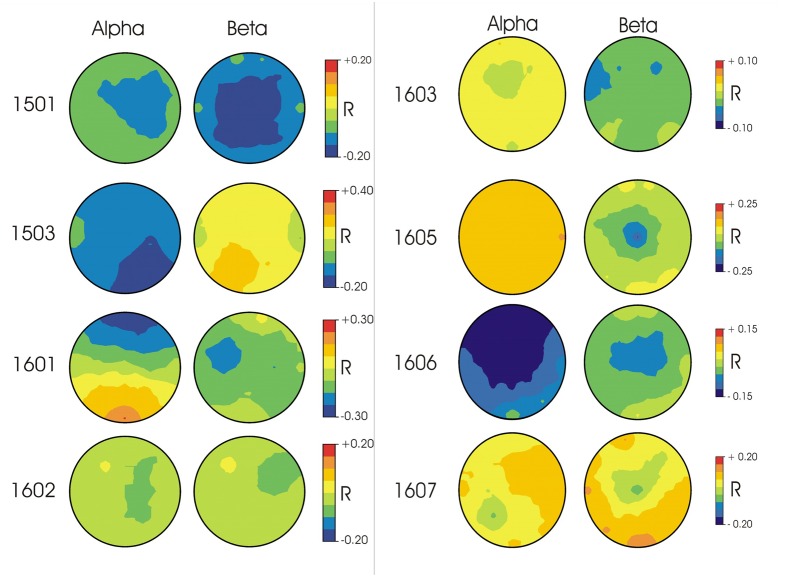
**EEG topographies for each participant of the correlation (Pearson’s *r*) between single trial EEG amplitudes in alpha (10–13 Hz) and beta (22–25 Hz) frequency ranges and response accuracy.** Blue indicates higher voltage associated with correct responses; Red indicates higher voltage associated with incorrect responses.

In terms of the topographies for specific frequency ranges, patterns differed widely across participants (**Figure 1**). For participant 1501, correct responses were associated with higher voltages in beta and alpha frequency ranges in widely distributed central regions. Participant 1503’s correct responses were associated with higher voltages in alpha frequency ranges and were strongest in posterior regions. For participant 1601, correct responses were paired with higher voltages in alpha frequency ranges in frontal regions as well as higher voltages in beta frequency ranges in left anterior areas. Participant 1602 showed weaker relationships between correct responses and spectral power, with stronger electrical activity in right anterior beta and more central regions for alpha frequencies. For participants 1603 and 1605, alpha frequencies demonstrated weaker correlations with correct responses than beta frequencies, with the strongest response in left anterior regions in the beta band for 1603 and central beta activity for participant 1605. Participant 1606 demonstrated the strongest correlation with correct responses within alpha frequencies in frontal/central regions. Participant 1607 showed no strong activity in either alpha or beta frequencies for correct responses.

## Discussion

The aim of this study was to determine whether spectral features obtained from EEG could be used to predict accuracy and error correction in persons with aphasia during a picture naming task. We found that for seven of the eight participants, EEG spectral features significantly correlated with accuracy (correct, incorrect) and error correction (correct, incorrect, self-corrected), although the correlations for error correction were weaker when error correction was used as a predictor. Furthermore, the predictive model was able to generalize (i.e., predict) participant responses in seven participants for accuracy and five for error correction. In terms of specific frequency bands, patterns of EEG activity varied greatly across individuals, with some participants showing stronger correlations with alpha activity, others showing stronger correlations with beta activity, and some showing weak correlations with both.

Our analyses were mainly concerned with developing models that could predict participant’s responses on individual trials with EEG features. Prediction models differ from models designed to test hypotheses (i.e., explanatory models) in several ways (Shmueli, 2010). Prediction models should be evaluated by cross-validation with data independent of that used for estimating model parameters rather than on the basis of the null hypothesis testing with the training data. In addition, issues related to characteristics of the predictors that would impact probability distributions (e.g., multivariate normality or multicollinearity) are not of concern for prediction models since the probability of the null hypothesis is not evaluated with the training data. Rather the concern is with the correspondence between predicted and observed data. Finally, interpretation of the coefficients of predictive models may be difficult due to multicollinearity (McFarland, 2013) and if explanation is the goal then the analyst is better advised to develop appropriate hypothesis testing models. However, in the present study we were concerned with models that predict participant performance on single trials as this is an initial step in developing a clinically useful tool.

Collectively, these results demonstrate that EEG spectral features can be used to reliably predict both the accuracy of a client’s response on a picture naming task and whether or not the client will correct the error, although the relationship between spectral features and error correction is not as reliable. This finding is in line with other studies that have been able to predict behavioral response from electrical activity in the brain (e.g., Klimesch et al., 1997; Osipova et al., 2006; Sederberg et al., 2007). In our experiment, we did not find a particular frequency range that was associated with accuracy or error correction; however, in examining the EEG response across the entire range of frequencies, the model could predict the response in a subsequent session. Now that we know that EEG error prediction is possible, we can use this information to design future studies that will more directly test this relationship (see, e.g., Burke et al., 2015; Salari and Rose, 2016). These possibilities include presenting naming trials only when task-appropriate activity is present, as in Salari and Rose (2016). Alternatively, clients could be trained to voluntarily generate task-appropriate activity in order to initiate naming trials in a manner analogous to that employed by McFarland et al. (2015) for a simple motor task.

Even though we were able to find significant correlations for most participants, one participant (1602) showed no significant correlations between EEG features and accuracy or error correction. What was different about this participant? He was right-handed with moderate transcortical motor aphasia. His time post-stroke onset was about 9 years, which was longer than any of the other participants by at least 5 years. Perhaps the amount of time post-stroke or the specific aphasia classification affected our ability to predict his responses. When examining overall accuracy and error patterns, there seemed to be nothing significantly different or notable. Whether time post-onset or aphasia classification is relevant variables remains a question for future studies.

For our initial analyses, we limited the number of EEG features in order to prevent overfitting. Thus, we included only three channels and six spectral bins. Although other channels and frequencies may well contain valuable information, at this early stage, we sought to be conservative. A subsequent analysis including nine channels did not improve performance during the test phase, possibly due to overfitting of the training data.

The high variability across participants suggests that our model’s ability to predict errors is limited to individuals and is unlikely to show the same EEG activity across different clients. In other words, an error prediction model must be tailored to the electrical signal of each individual. One could imagine an application of this statistical model in the therapy room, which might involve acquisition of an initial EEG “signature” for an individual by administering a baseline task prior to initiation of therapy. Using the client’s individualized EEG “signature” of error production, a clinician could then monitor the EEG signal during the session and be alerted when the client is likely to produce an incorrect response.

The impact of such a therapy tool could be great, especially when considering its potential in the context of errorless learning and retrieval practice. As briefly reviewed in the introduction, results from studies investigating outcomes of these two training approaches are mixed. In general, when these approaches are applied to naming treatments for persons with aphasia, errorless learning has been demonstrated to be at least as effective as errorful learning and tends to be preferred by clients with aphasia (Fillingham et al., 2006). Others have found that errorless learning is more effective in the short term, whereas retrieval practice is more effective in the long term (Middleton et al., 2015). Development of an individualized error prediction model would provide a potential way to merge the two treatment approaches. If clinicians can predict when a client will produce an error before he/she attempts the production, this may help to avoid the potentially detrimental effects of error learning while still receiving the long-term benefits of retrieval practice. Some have suggested that feedback during therapy is the key to success (e.g., McKissock and Ward, 2007), and others have shown that specific types of cueing are more likely to lead to error learning (Middleton and Schwartz, 2013). Using an error prediction model in the therapy session would also potentially allow clinicians to provide cueing that is tailored to the individual to optimize the learning experience.

Although the findings from this study are encouraging and provide preliminary evidence that EEG spectral features can be used to predict response accuracy in persons with aphasia, the study is not without limitations. The participants in this study represented a wide range of aphasia severities and subtypes, which could have limited the strength of our findings. Our rationale for the inclusion of such variability in our sample was that our study was intended to be a preliminary investigation of future possibilities. Now that we have some evidence that EEG spectral features can be predictive of response accuracy, future studies can more carefully control participant selection. It is possible, for example, that certain aphasia profiles are more ideal than others for error prediction (e.g., different results for participant 1602). As is an issue in many aphasia studies, this study had a relatively small sample size, although our analysis focused on individual participants, making sample size less relevant than for studies that examine group differences. Another area that requires more investigation is the reliability of error prediction using this model. For this study, we only compared the model’s predictions with one session of observed scores for each participant. In order to further refine and test the prediction model, prediction reliability will need to be tested more rigorously, particularly before it can be applied in a clinical setting. While our initial results were largely positive, further research will likely produce improved prediction performance. For example, many potential EEG features could be explored. These include not only amplitude features at additional channels but also phase effects; however, exploration of these possibilities awaits larger data samples.

## Conclusion

We have demonstrated evidence that an individual’s EEG spectral features can reliably predict response accuracy on a picture naming task. This is an essential first step toward developing a clinically useful tool for predicting errors in speech and language therapy and possibly bridging the gap between errorless learning and retrieval practice approaches in naming therapy.

## Ethics Statement

This study was approved by the Syracuse University Institutional Review Board. Participants were provided with a written consent form and a member of the research team verbally reviewed the details of the study and answered any questions. The participant was provided with a copy of the signed consent form and reminded of his/her right to withdraw from the study at any time without penalty. Given the strong desire many individuals with stroke and aphasia have to recover, it is critical that these individuals understand their participation in this research study does not constitute therapy. All personnel involved with consenting participants was specifically trained to discuss the details of the study and make clear to the participants that enrolling in the research study will not directly help with their recovery.

## Author Contributions

ER contributed to the research design, acquisition, analysis, and interpretation of data, and the writing of this manuscript. DM contributed to the research design, analysis and interpretation of data, and the writing of this manuscript. Both authors agree to be accountable for all aspects of the work.

## Conflict of Interest Statement

The authors declare that the research was conducted in the absence of any commercial or financial relationships that could be construed as a potential conflict of interest.
